# Narrative review of the scientific and methodological explanations for 101 mechanisms of opioid tolerance

**DOI:** 10.1097/PR9.0000000000001357

**Published:** 2025-11-14

**Authors:** Michael M. Morgan, Erin N. Bobeck

**Affiliations:** a Department of Psychology, Washington State University Vancouver, Vancouver, WA, USA; b Department of Biology, Interdisciplinary Doctoral Program in Neuroscience, Utah State University, Logan, UT, USA

**Keywords:** Antinociception, Analgesia, Morphine tolerance, Pain

## Abstract

Opioids such as morphine and fentanyl are widely prescribed treatments for pain. Unfortunately, opioid use is limited by side effects, dependence, and the development of tolerance with repeated use. A major goal of preclinical opioid research is to identify the neural mechanisms underlying tolerance to opioids to provide better pain management and reduce side effects caused by high opioid doses. To that end, preclinical pain researchers have reported well over 101 mechanisms of opioid tolerance. The objective of this review is to describe both the scientific explanations for multiple mechanisms of opioid tolerance (eg, different types of opioid tolerance, different mechanisms at different locations and for different opioids) and the methodological problems that cause false positives. For example, many changes correlate with but do not cause tolerance. Opioid binding to mu-opioid receptors (MOR) activates a signaling cascade that includes numerous molecules and cells making it difficult to identify the precise mechanism causing tolerance. Other problems, such as lack of appropriate control groups or confounds produced by antinociceptive or motor effects of the treatment, are surprisingly common. Solutions to these problems are presented at the end of the article in the hope of guiding future research. Although the focus is on opioid tolerance, the problems and solutions presented here apply to tolerance to other drugs and any neural change that occurs over time such as learning.

## 1. Introduction

Opioids are commonly used for the treatment of pain, but their use is limited by side effects, dependence, and the development of tolerance. A significant amount of preclinical pain research has focused on trying to understand the neural mechanisms underlying opioid tolerance (Table 1). Drug tolerance is defined as a decrease in potency with repeated administration. This decrease in potency requires increasing the dose to maintain analgesic efficacy. Prolonged administration of high opioid doses facilitates the development of dependence and exacerbates the occurrence of unpleasant and dangerous side effects. An understanding of the mechanisms underlying opioid tolerance holds the promise of facilitating the development of treatments to disrupt this process. The clinical relevance of the vast preclinical literature examining the mechanisms of opioid tolerance depends on the validity of these experiments. This review provides a critical analysis of this preclinical research.

**Figure 1.**
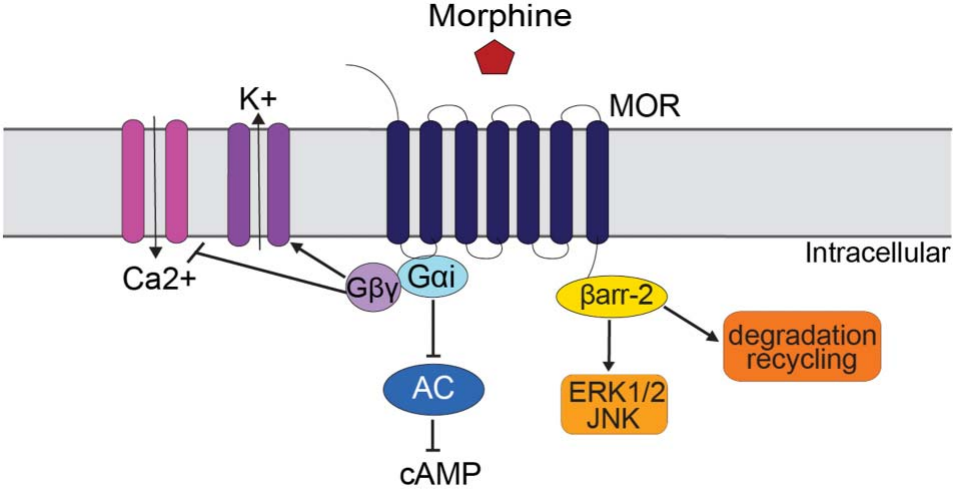
Schematic of traditional mu opioid receptor (MOR) signaling pathways. Activation of the MOR with an opioid such as morphine inhibits neural activity via G protein signaling. These G-proteins inhibit adenylyl cyclase (AC), close calcium (Ca2+) channels, and open potassium (K+) channels. Some opioids also activate β-arrestin (ßarr-2) which triggers receptor trafficking to the intracellular space and shifts signaling from G proteins to mitogen-activated protein kinases (ie, ERK1/2 or JNK). The MOR is then either degraded or recycled back to the plasma membrane. ERK1/2, extracellular regulated kinase 1/2; JNK, cJun N-terminal kinase.

Opioids such as morphine, fentanyl, and oxycodone produce antinociception by binding to mu-opioid receptors (MOR). Mu-opioid receptors are G protein-coupled receptors (GPCR) linked to multiple intracellular signaling pathways (Fig. 1) that alter neural activity, gene expression, and neuron/glia communication. Tolerance to opioids could be mediated by changes in intracellular signaling, gene expression, glial signaling, interactions with other neurons, or a combination of these mechanisms.^118^

**Table 1:** Putative mechanisms of morphine tolerance.

1. Mast cell degranulation of RBL-2H3 cells^126^
2. CB2 receptor activation on sensory afferents^18^
3. G protein-coupled estrogen receptor activation of on-cells^51^
4. Increased release of cholecystokinin (CCK)^46^
5. Presynaptic MOR endocytosis by GRK phosphorylation^56^
6. Ptchd1-mediated MOR endocytosis via cholesterol regulation^81^
7. Delta opioid receptor signaling^50^
8. nPAS domain protein 2 in fentanyl tolerance in females^101^
9. Expression of receptor transporter protein 4 (RTP4)^35^
10. Upregulation of VGluT2^43^
11. Activation of BDNF/TrkB pathway^43^
12. Activation of angiotensin II receptor type 1^155^
13. Inhibition of the endocannabinoid degradation (FAAH)^8^
14. ABIN-1 regulation of MOR internalization^153^
15. P2X4 receptors in medial prefrontal cortex^150^
16. Porcine diazepam binding inhibitor (pDBI)^102^
17. Increased expression of TCF7L2^20^
18. Inhibition of TAK1 phosphorylation^133^
19. Activation of 5-HT3 receptors^106^
20. Platelet derived growth factor receptor ß (PDGFRß)^71^
21. E3 ubiquitin ligase Pellino1 (Peli1) in tolerance development^134^
22. NLRP3 expression^76^
23. Epidermal growth factor receptor (EGFR) signaling^100^
24. Hyperpolarization-activated cyclic nucleotide-gated channels^146^
25. Upregulation of MOR 1B2 and MOR 1C1^19^
26. Shift in MOR 1B2 and MOR 1C1 from Gi/Go to gs^19^
27. Increased Notch 1 signaling^105^
28. Decreased histone deacetylase (HDAC)-1^105^
29. Downregulation of MOR mRNA^9^
30. Decreased AMPK^110^
31. Phosphorylation of S6K1^111^
32. Phosphorylation of 4E-BP1^111^
33. Increased mitochondrial calcium uniporter (MCU)^120^
34. Increased CREB^120^
35. Reduced PINK1/Parkin clearance of damaged mitochondria^60^
36. Primary afferent activation of NMDA receptors^30^
37. Reduced AMPA receptor signaling^47^
38. Vasopressin 1b receptor signaling^61^
39. Increased PKC^49^
40. Increase in M2 spinal microglia^54^
41. Increased ß-ARK1 regulation of NMA receptors^152^
42. P2X7 receptor activation of microglia^66^
43. Decreased presynaptic GABA vesicles^140^
44. Downregulation of microRNA miR-375^69^
45. Increased BDNF production^69^
46. Constitutive MOR trafficking^82^
47. Hypermethylation of the MOR gene OPRM1^130^
48. Orexin activation of OX1 receptors^84^
49. Increased CXCL12^74^
50. Toll-like receptor 4 (TLR4) neuroinflammation^32^
51. Increased tumor necrosis factor (TNF)^32^
52. MOR on primary afferents^26^
53. Increased expression of CXCR10 in microglia^135^
54. JNK2 signaling^147^
55. T394 phosphorylation of the MOR^136^
56. Upregulation of microRNA miR-212/132^38^
57. Upregulation of heat shock protein 27 (HSP27)^75^
58. Decreased imidazoline receptor antisera-selected (IRAS)^68^
59. Upregulation of GluN1 subunit mRNA^151^
60. Activation of ß-arrestin-2^25^
61. Activation of spinal glial cells^33^
62. Upregulation of MOR splice variant MOR-1B2^129^
63. Upregulation of MOR splice variant MOR-1C1^129^
64. Decrease in neurogenic differentiation 1 Neurod1^70^
65. Activation of mTORC1^143^
66. Increased thrombospondin 2 (TSP2)^98^
67. Adrenomedullin activation of microglia^149^
68. Upregulation of microRNA miR-339-3p^141^
69. Activation of MOR interacting proteins^97^
70. Activation of dopamine D2 receptors^93^
71. Decrease MOR gene expression^17^
72. Decrease NOP gene expression^17^
73. BDNF release^80^
74. Formation of MOR/DOR heterodimer^119^
75. Increased nitric oxide^77^
76. Decreased bovine adrenal medulla 22 (BAM22)^132^
77. Activation of soluble guanylate cyclase (sGC)^92^
78. Neuronal apoptosis^42^
79. Decreased sensitivity of presynaptic MORs^36^
80. Nigella sativa oil inhibition of nitric oxide^1^
81. Upregulation of Ca++/calmodulin-dependent protein kinase II^21^
82. P2X7 receptors^156^
83. Shift in MOR signaling from G_s_ to G_i_^55^
84. Activation of phosphodiesterase^73^
85. Upregulation of microRNA miR-23b^142^
86. Calcitonin gene-related peptide (CGRP)^137^
87. Imidazoline receptor inhibition of MOR internalization^37^
88. Increase in interleukin-6 (IL-6)^48^
89. Increased p38 MAPK^28^
90. Increase in interleukin-1 (IL-1)^109^
91. Activation of L-type Ca++ channels^31^
92. Activation of R-type Ca++ channels^145^
93. Activation of CXCR3 receptors^53^
94. Activation of the efflux transporter P-glycoprotein (P-gp)^5^
95. Activation of phospholipase C (PLCß3)^114^
96. Increased nociception/orphanin FQ^124^
97. Activation of imidazoline receptor^59^
98. Neuropeptide FF^64^
99. Activation of melanocyte inhibiting factor (MIF-1)^148^
100. Cyclic adenosine monophosphate^27^
101. Adenylyl cyclase activation^108^

This table does not include a comprehensive list of reported mechanisms of opioid tolerance. The search was conducted in PubMed and ended in 2023 when 101 mechanisms were reached.

Despite thousands of studies on opioid tolerance, a clear understanding of the mechanisms underlying opioid tolerance remains elusive. The title of this article claims there are 101 mechanisms of opioid tolerance. To be true to the title, Table 1 lists 101 mechanisms. This is not a comprehensive list but was compiled to make a point. The fact that over 101 mechanisms of opioid tolerance have been reported is shocking and suggests a level of complexity that makes a farce of Occam's Razor. Although it is unlikely there is a single mechanism of opioid tolerance, it also is unlikely there are over 101 distinct mechanisms. The 3 objectives of this review are to (1) describe the scientific reasons for multiple mechanisms of opioid tolerance; (2) analyze the methodological problems that result in false positives; and (3) outline a methodology to more accurately identify the mechanisms underlying opioid tolerance.

## 2. Scientific reasons for multiple mechanisms of opioid tolerance

There are 4 scientific reasons for multiple mechanisms underlying tolerance to opioids. First, there are multiple types of opioid tolerance, and each type engages a unique mechanism. Second, opioids bind to receptors throughout the nervous system and the specific mechanism of tolerance varies by neuron, location, and circuit. A third reason is that different opioids interact with opioid receptors in different ways leading to different mechanisms of tolerance. And finally, MOR signaling varies depending on which molecules and receptors are also present in the cell.

### 2.1. Types of opioid tolerance

Most of the mechanisms of tolerance listed in Table 1 are the result of putative changes in neural signaling caused by receptor activation. This type of tolerance, caused by dynamic changes in neurons as a result of opioid binding to MORs, is known as pharmacodynamic tolerance. Neural adaptations can also occur independent of opioid actions in a process known as Behavioral Tolerance. In addition, pharmacokinetic tolerance occurs when bodily adaptations reduce the amount of opioid gaining access to opioid receptors.

Table 1 provides a long and diverse list of putative pharmacodynamic mechanisms of opioid tolerance. These adaptations are triggered by opioid receptor binding which triggers negative feedback mechanisms to reduce signaling from subsequent opioid administration. The simplest mechanism is receptor down-regulation as described in early studies of opioid tolerance.^122,144^ More recent studies focus on receptor trafficking or changes in downstream intracellular signaling molecules.^10,40,52^ Experiments examining the mechanisms underlying pharmacodynamic tolerance are technically challenging (see Section 4) and often methodologically flawed (see Section 3).

Pharmacodynamic tolerance can be divided into Associative and Non-Associative mechanisms. Intrinsic neural adaptations, as described in the preceding paragraph, is nonassociative. Associative tolerance refers to the influence of learning mechanisms that alter the effects of the opioid. For example, cues that predict opioid administration alter how neurons respond to the opioid. The profound impact of associative tolerance is highlighted by the groundbreaking research of Shepard Siegel in the 1980s. This research, conducted in both rats and humans, showed that removal of environmental cues associated with opioid administration reduced tolerance leading to drug overdose.^112,113^ Although Siegel did not assess the neural mechanism underlying associative tolerance, interactions between neurons processing sensory information and opioid effects must be involved. Anytime opioids are administered repeatedly, that is with cues, the potential for engaging associate mechanisms of tolerance is present.

**Figure 2.**
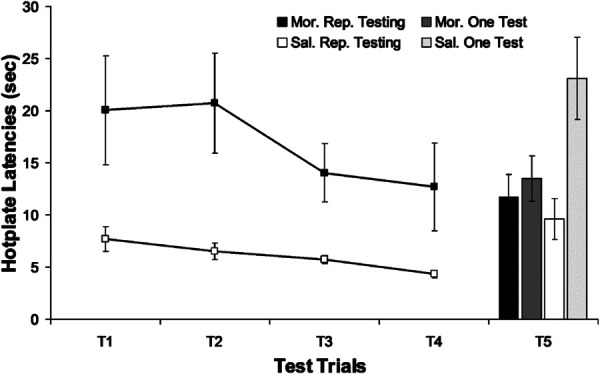
Data from Lane and Morgan, 2005,^65^ showing behavioral tolerance. Rats received daily injections of morphine or saline and were either tested using the hot-plate test each day or returned to their home cage with no testing. Trials 1–4 (T1, T2…) show a gradual reduction in hot-plate latency in both morphine and saline treated rats. All 4 groups were injected with morphine on trial 5. The antinociceptive effect of morphine was reduced in rats that received repeated injections of morphine and in those that were repeatedly tested. The bar graph on the right shows that repeated hot plate testing in rats injected with saline showed significantly less antinociception to morphine than saline treated rats that had not been undergone repeated testing.

Behavioral tolerance refers to adaptations that occur with repeated testing. Repeated nociceptive testing enhances an animal's response to a noxious stimulus, and this change has been shown to reduce the antinociceptive effect of morphine.^65^ This adaptation could be caused by learning to respond to avoid the noxious stimulus, a reduction in stress from repeated testing, or some other unknown mechanism. Whatever the cause, behavioral tolerance is a common confound in studies of pharmacodynamic mechanisms of tolerance because many experiments test animals every day to demonstrate the progressive development of tolerance (Fig. 2). The obvious solution, as described in section 4.3 is to avoid repeated testing.

Pharmacokinetic mechanisms of tolerance are potentially engaged whenever an opioid is administered. In contrast to how opioids alter the activity of neurons as occurs in pharmacodynamic mechanisms of tolerance, pharmacokinetic mechanisms focus on how the body changes the drug. The most common pharmacokinetic mechanism of tolerance is enhanced opioid metabolism with repeated administration.^89,96^ However, other changes such as increased plasma protein binding, increased elimination, or decreased blood brain barrier transport could also contribute. The net impact is a reduction in opioid levels at the receptor. No neural mechanism is engaged by pharmacokinetic tolerance, but the net effect is a reduction in antinociception with repeated administration.

### 2.2. Location of opioid receptors

Mu-opioid receptors are located throughout the central nervous system,^62,78^ providing opportunities for multiple mechanisms of opioid tolerance. Opioid receptors located on cells in the periphery^23^ are also sensitive to the development of tolerance with repeated opioid administration.^3,45^ The degree to which neural mechanisms for tolerance are conserved across binding sites is not known, but different mechanisms at different neural locations have been reported. For example, glutamate signaling has been reported to contribute to tolerance at the spinal level,^127^ but not at the supraspinally located periaqueductal gray.^85^ Moreover, the ventrolateral periaqueductal gray (PAG) is very sensitive to the development of tolerance to direct morphine administration,^12,87^ whereas adjacent structures such as the lateral PAG, dorsal raphe nucleus, and rostral ventromedial medulla are not.^16,86,125^ Opioids administered systemically will bind to opioid receptors throughout the nervous system and thus engage multiple mechanisms of opioid tolerance. Moreover, opioids bind different types of receptors (eg, mu- and delta-receptors) and alter the activity of a wide range of cells (eg, neurons and glia) within a single structure, resulting in multiple potential tolerance mechanisms within a specific location.

### 2.3. Different mechanisms for different opioids

A third reason for multiple mechanisms of opioid tolerance is that different mechanisms are engaged by different opioids. Although opioids produce analgesia via activation of MORs, ligand-biased signaling shows that not all opioids interact with the MOR in the same way. High efficacy agonists such as fentanyl activate ß-arrestin signaling and receptor internalization, whereas the primary signaling pathway for lower efficacy agonists such as morphine is inhibition of adenylyl cyclase via G-protein signaling^104,138^ (Fig. 1). These differences in signaling result in opioid-specific mechanisms of tolerance. For example, G protein-coupled receptor kinase (GRK) signaling has been shown to contribute to fentanyl tolerance, whereas cJun N-Terminal Kinase (JNK) signaling contributes to morphine tolerance.^83,88^ The lack of cross tolerance between morphine and fentanyl microinjections into the ventrolateral PAG confirms the engagement of different mechanisms of tolerance for fentanyl and morphine.^14^ In addition, the high efficacy agonist etorphine downregulates MORs, an effect not seen with repeated morphine administration.^116^ These data demonstrate that the mechanism for opioid tolerance may differ even when 2 opioids bind to the same receptor in the same brain structure.

### 2.4. Opioid receptor dynamics

Mu-opioid receptor signaling is a necessary step in the development of pharmacodynamic tolerance. This signaling, and thus the mechanism underlying tolerance, can vary depending on factors affecting the MOR. First, there are many MOR variants.^95^ As would be expected, these receptor polymorphisms can alter both opioid signaling^103^ and the mechanism underlying tolerance.^94^ Second, MORs are found at both presynaptic and postsynaptic sites.^24,128^ In vitro experiments have revealed a number of putative molecular mechanisms of tolerance (eg, MOR desensitization, internalization, adenylyl cyclase upregulation) linked to postsynaptic MORs,^139^ but some of these mechanisms, such as MOR desensitization, do not appear to contribute to tolerance mediated by presynaptic MORs.^36^ Third, the molecular environment surrounding MORs also alters signaling. Both the presence of membrane cholesterol^63,67^ and the formation of receptor oligomers^34,117^ modify MOR signaling. The formation of a mu/delta receptor heterodimer is a putative mechanism underlying opioid tolerance.^41,99^ Oligomers could also contribute to opioid tolerance by opening novel signaling pathways.^34,117^

## 3. Methodological problems

Despite legitimate scientific reasons for multiple mechanism of opioid tolerance as described in Section 2, experiments examining the neural mechanisms underlying tolerance are methodologically challenging, resulting in what are probably many false positives. In some cases, the reported mechanism correlates with but does not cause tolerance. In other cases, methodological problems result in falsely attributing a change in antinociception to inhibition of opioid tolerance. These false positives could result from the use of reduced preparations, time and testing confounds, manipulations that alter nociception instead of tolerance, and the use of drugs that block the opioid from engaging the tolerance mechanism. Each of these problems are discussed below.

### 3.1. Correlation ≠ causation

One approach in trying to identify specific neural mechanism for opioid tolerance is to conduct a correlational study. This approach can only eliminate potential mechanisms because signaling pathways that do not change cannot cause tolerance. The problem is that opioid administration causes thousands of changes in the nervous system because opioid receptors are widely distributed and activation of these receptors will result in inter- and intracellular changes. Many genomic,^123^ proteomic,^57,72^ and electrophysiological^4,44,154^ changes are evident following a single morphine injection. Genes, proteins, and neurons that change following such an injection may contribute to tolerance, but most of these changes are merely correlational and not the cause of opioid tolerance. All signaling mechanisms downstream from the change that underlies tolerance will change as tolerance develops. These changes are also correlational, not causal. The many changes caused by opioid administration provide so many distractions that it is difficult to identify actual mechanisms.

### 3.2. Problems with reduced preparations

Reduced preparations such as cell culture and in vitro slice recordings are extremely valuable in revealing signaling mechanisms. Much of what is known about opioid signaling is based on such experiments. Reduced preparations have been used to reveal a number of putative mechanisms of opioid tolerance.^2^ These mechanisms are putative in the sense that drug tolerance is defined as a change in behavior with repeated administration. The many molecular changes reported with in vitro experiments^139^ must be confirmed by testing these mechanisms in behaving animals.

Immortalized cell lines such as HEK-293 and CHO cells are very useful in answering specific intracellular signaling questions. Transfection of proteins into the cell allows specific signaling mechanisms to be studied. Signaling changes produced by prolonged opioid administration could reveal potential mechanisms underlying tolerance. The concern is whether the adaptations revealed by such experiments are the same as occur with repeated opioid use in naturally occurring neurons in an intact animal. For example, administration of a metabotropic glutamate receptor 5 (mGlu5) antagonist inhibited opioid-induced phosphorylation, internalization, and desensitization of MORs in HEK-293 cells.^107^ This change may contribute to opioid tolerance or it may simply be an epiphenomenon of a “Frankenstein” cell selectively transfected with glutamate and opioid receptors. Overexpression of mGlu5 receptors and other proteins in transfected cells raise questions about the biological relevance of the signaling. In vivo administration of the same mGlu5 receptor antagonist has been reported to block opioid tolerance,^39,115^ but this effect is probably the result of enhanced opioid antinociception^91,157^ not tolerance specifically (see Section 3.3.2). The main point is that attributing changes in mGlu5 receptors or any other mechanism to opioid tolerance is not possible by analysis of transfected cells alone.

In vitro slice recordings provide a more natural preparation to identify potential signaling adaptations produced by opioids. Much has been revealed about opioid signaling from such recordings. Removing a slice of tissue disconnects neurons from input and output circuits but preserves local connections and neuronal function. Analysis of specific neurons in such a slice have revealed MOR desensitization and internalization following infusion of an opioid into the recording bath.^7,79,90^ The debate about whether MOR receptor internalization contributes to^6^ or prevents the development of tolerance^58^ highlights the correlational nature of such experiments.

Another problem with in vitro experiments is that the timing for desensitization seems incompatible with the tolerance that develops in intact animals. Bath application of opioids produces receptor desensitization that is evident within 5 minutes and persists for approximately 45 minutes following termination of administration.^131^ In contrast, the tolerance that occurs in laboratory animals can occur after a single opioid injection^12^ but typically requires prolonged or repeated administration and results in reduced opioid efficacy that can persist for days.

One method to link the behavioral effects of opioids to cellular mechanisms is to induce tolerance with prolonged in vivo administration and then assess changes in specific tissues.^22,29,36^ This approach does not allow analysis of changes to specific neurons over time because the in vitro cellular recording occurs following euthanasia. However, comparison of neurons from opioid tolerant and control animals can reveal differences at the cellular level. The assessment of both cellular and behavioral changes is a useful methodology in identifying potential mechanisms of tolerance. Nonetheless, such data are correlational and require subsequent experiments blocking specific signaling mechanisms to move to a causal understanding of tolerance.

### 3.3. Problems with in vivo experiments

Ultimately, all mechanisms of opioid tolerance must be studied in whole animals. Such experiments are almost impossible to conduct in humans because of ethical concerns about opioid administration and treatment. Animal research provides much greater control if done correctly. Unfortunately, many experiments are not done correctly. Many of the reported mechanisms of tolerance could be the result of experimental confounds. Confounds are difficult to control because tolerance develops over time and opioids produce many changes. The difficulty in designing experiments to reveal the neural mechanisms underlying opioid tolerance are evident by examining common design problems.

#### 3.3.1. The confound of time

Any experiment that requires repeated or prolonged drug administration is confounded by time. Time alone is typically not a problem, but changes in response to an injection or repeated testing over time make it difficult to distinguish between behavioral and pharmacodynamic tolerance or associative and non-associative tolerance. The best control for time-related changes is to include a nonopioid treated control group. Surprisingly, many experiments do not include such a control. A nonopioid treated control group receives the same number of injections and nociceptive tests as the opioid treated group but only receives the opioid on the last day of testing. A greater antinociceptive effect in the control group compared with the opioid treated group would indicate pharmacodynamic tolerance. Comparable antinociception in both groups would indicate Behavioral Tolerance as discussed above (Fig. 2 and Section 2.1).

#### 3.3.2. Treatments that have antinociceptive effects

Treatments that block opioid tolerance are defined by their ability to reinstate opioid antinociception. A problem occurs when a treatment produces antinociception independent of the opioid or in synergy with the opioid. In either case, administration of the “treatment” and the opioid produces antinociception. Such an effect looks like reversal of opioid tolerance but blocking tolerance and enhancing antinociception are distinct effects. The obvious solution is to assess the antinociception effects of the treatment alone and in combination with the opioid in nontolerant animals. If acute administration of the treatment enhances opioid antinociception, then any reported reversal of opioid tolerance can be explained as drug synergy. Few experiments assess the antinociceptive efficacy of the treatment. However, even in experiments where antinociceptive effects of the treatment are reported, this antinociception is often discounted by concluding that the treatment reversed opioid tolerance.^11,15,157^

#### 3.3.3. Treatments that have motor effects

Similar to the antinociceptive effect described above, treatments that have sedative, paralytic or other disruptive effects can also reduce the response to a noxious stimulus. This lack of response may be interpreted as antinociception or reversal of opioid tolerance. Many studies address this potential problem by using a treatment dose that has no effect by itself. However, the combination of a low treatment dose with an opioid can produce synergistic effects. The only way to avoid this confound is to assess antinociceptive tolerance after both drugs are no longer present in the animal's body (see Section 4.4). Most drugs have a short enough half-life that this test can be conducted on the day following the last injection.

#### 3.3.4. Treatments that block antinociception

**Figure 3.**
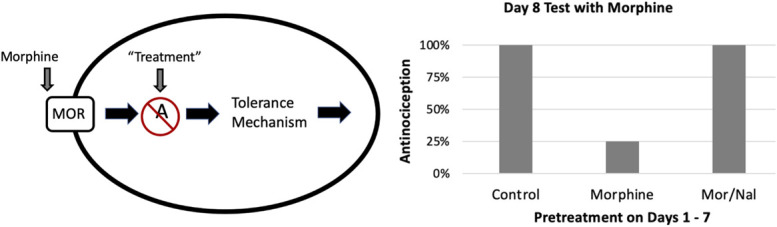
Model of a neuron and proposed data in which the putative treatment prevents engagement of the tolerance mechanism. Blocking the MOR with naltrexone or a treatment that blocks early signaling (eg, A) would prevent engagement of the tolerance mechanism. The expected results of such an experiment are shown on the right. Control rats injected with vehicle for 7 days will show a full antinociceptive effect when injected with morphine on day 8. Rats given morphine on days 1–7 will be tolerant showing no antinociception to the opioid on day 8. Coadministration of naltrexone and morphine on days 1–7 will prevent engagement of the tolerance mechanism, so administration of morphine on day 8 will produce a full antinociception effect (Mor/Nal).

Opioid tolerance is caused by an adaptation in the signaling cascade following opioid binding to a MOR. Signaling begins at the MOR and ends with a change in an ion channel which determines the release of neurotransmitter (Fig. 1). Tolerance can occur by a change anywhere along this signaling cascade or as a change in signaling between cells. Any treatment that blocks signaling before the tolerance mechanism will appear to block tolerance but merely prevents the tolerance mechanism from being engaged. The most obvious example of this is coadministration of morphine and the opioid receptor antagonist naltrexone. Coadministration of these drugs for 7 days would prevent morphine from engaging the mechanisms that underlie tolerance. However, the antinociceptive effect produced by injecting morphine on day 8 would look like the previous naltrexone injections blocked the development of tolerance (Fig. 3).

Administration of naltrexone is an extreme example, but any treatment that prevents the opioid from engaging the tolerance mechanism would have the same effect. The difference between blocking opioid antinociception and opioid tolerance can be determined by assessing the effect of the treatment on opioid antinociception in nontolerant animals. A drug that selectively blocks the adaptation underlying opioid tolerance should have no effect on the acute antinociceptive effect of opioids. Identifying the pathway that leads to tolerance will ultimately lead to the tolerance mechanism, but the drugs that block these steps are not useful as treatments because they will block the desired analgesic effect.

## 4. Proper experimental design

Experiments examining the mechanisms underlying opioid tolerance are complicated because 2 drugs, an opioid and the treatment, are administered over time. Two drugs can interact in many ways, and time is an unavoidable confound. Tolerance experiments are further complicated by the fact that the mechanism for tolerance is embedded in a signaling cascade that includes multiple molecules and cells. These challenges highlight the special care needed in designing experiments to reveal the mechanisms underlying opioid tolerance. That said, the only thing unique about the experimental design approaches described below is how rarely they are implemented.

### 4.1. Control groups

All well-designed experiments include a nontreated group to control for extraneous variables. Thus, it is surprising how rarely all 3 control groups are included in tolerance experiments. Experiments examining treatments for opioid tolerance require a control group for the treatment, another for the opioid, and a third group that receives neither the treatment nor the opioid. This results in a 2 × 2 experimental design with 4 conditions (Table 2). Comparison of the opioid alone group to the nondrug control group will reveal the magnitude of tolerance. The treatment/opioid group will reveal the degree to which the treatment reverses opioid tolerance, and the treatment alone group will reveal whether the treatment has antinociceptive or motor effects independent of opioid tolerance. These effects are only revealed when the opioid is administered to all 4 groups after the treatment has cleared the system (This methodological approach is discussed in more detail in Section 4.4).

**Table 2:** Required experimental and control conditions.

	Treatment	Vehicle
Morphine	Reversal of tolerance	Tolerance
Vehicle	Side effects/antinociception	No tolerance

### 4.2. Dose response analysis

**Figure 4.**
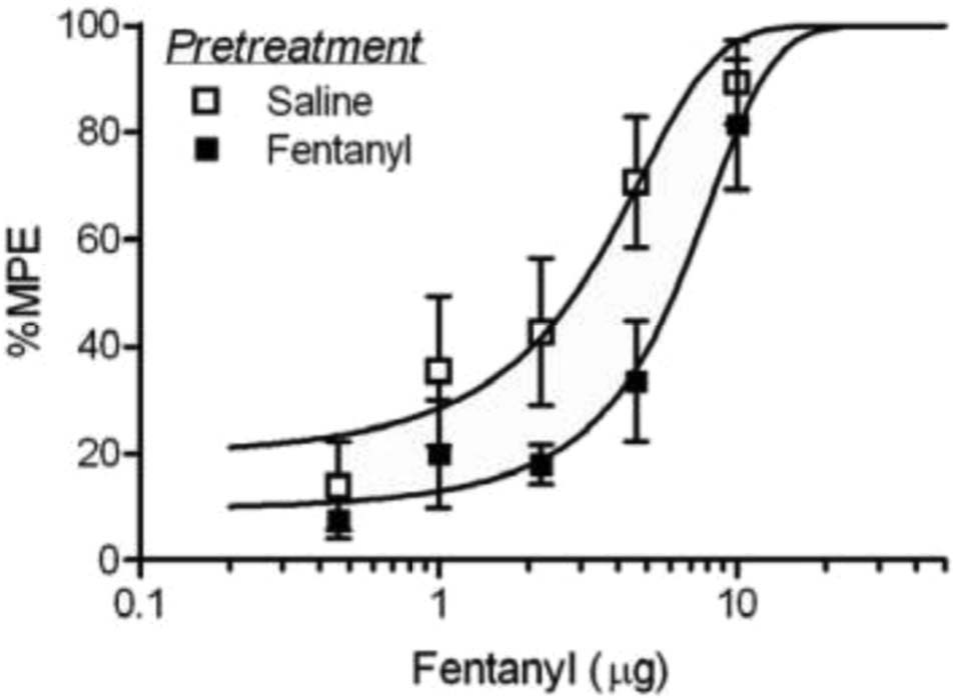
Tolerance to repeated injections of fentanyl caused a rightward shift in the dose-response curve. Rats received 2 injections/day of saline or fentanyl for 4 days. Both groups received third log doses of fentanyl on day 5 to assess tolerance (Fig. 7 in Ref. 12).

The pharmacological definition of tolerance is a rightward shift in the dose-response curve. Log step doses that cover the full antinociceptive range produce an S-shaped dose-response curve where the curve flattens at the top to reveal drug efficacy and the dose that corresponds to half the maximal effect (ED50) reveals drug potency.^121^ Given that opioids tend to have a steep dose-response curve, we use quarter-log (1, 1.8, 3.2, 5.6, and 10 µg) or third-log (1.0, 2.2, 4.6, and 10 µg) doses.^13,87^ An opioid tolerant animal will have a reduced antinociceptive effect at each dose along this curve, resulting in what appears as a rightward shift in the dose-response curve (Fig. 4). The experimental question is whether this decrease in antinociceptive potency is reversed by the treatment. The problem with assessing tolerance with a single dose as done in many experiments is that the selected dose could be submaximal or supramaximal preventing assessment of a change in effect.

Most experiments repeatedly administer a supramaximal opioid dose to induce tolerance. The dose-response assessment should be conducted at the end of this opioid administration protocol. A different group of animals can be used to test each dose, but such an approach requires a lot of animals. This number can be reduced by using a cumulative dosing method.^87^ Cumulative dosing uses a within-subjects design in which animals are tested approximately every 20 minutes following increasing opioid doses that when added together produce log steps. An example with half-log doses would be to inject animals with 1.0 mg/kg of morphine and test them 20 minutes later. A second injection of 2.2 mg/kg would occur immediately after this test, and then a final injection of 6.8 mg/kg after the second test to produce cumulative half-log doses of 1, 3.2, and 10 mg/kg. The delay between injections is a potential problem with cumulative dosing, but the savings in the number of animals and time far outweigh this cost. We have seen consistent rightward shifts in the dose response curve when repeatedly administering opioids using a cumulative dosing procedure on the test day.^12,13^

### 4.3. Limit antinociceptive testing

As described above, assessment of tolerance is conducted on the last day using a dose-response analysis. Testing animals daily to show the gradual reduction in antinociception is common, but misguided. Repeated testing in the absence of opioid administration produces Behavioral Tolerance (see Section 2.1, Fig. 2), which confounds assessment of opioid-induced changes. Behavioral Tolerance is easily avoided by injecting the opioid in the absence of any behavioral testing except on the final day when tolerance is assessed by comparing the effect of the opioid in all animals (Table 3). Ideally, an initial experiment in drug-naïve animals would assess the main effects and interactions between the opioid and treatment. Such an experiment can reveal the appropriate dose to use and whether the treatment enhances or reduces the effect of the opioid independent of the development of tolerance (see Sections 3.3.2–3.3.4).

**Table 3:** Two-step process to evaluate a novel treatment for opioid tolerance.

Experiment 1: acute assessment of opioid/treatment main effects and interactions	
Drug administration	Tests
Opioid and treatment (vehicle, low, medium, high doses)	Antinociception and motor effects
Vehicle and treatment (vehicle, low, medium, high doses)	Antinociception and motor effects

Experiment 2: impact of treatment on the development of opioid tolerance	
Days 1–5 injections, no testing	Day 6 injections and tests
Vehicle/vehicle	Opioid dose-response: assess antinociception
Vehicle/treatment	Opioid dose-response: assess antinociception
Opioid/vehicle	Opioid dose-response: assess antinociception
Opioid/treatment	Opioid dose-response: assess antinociception

### 4.4. Development vs expression of tolerance

A first step in any experiment examining the mechanism for tolerance is to decide whether the goal is to block the development or expression of tolerance. Experiments focused on the development of tolerance require coadministration of the putative treatment before or concomitant with each opioid injection. Tolerance is assessed by injecting all rats with the opioid after the last treatment to avoid treatment-induced confounds (eg, antinociception, motor effects). All 4 experimental groups would receive log-step doses of the opioid, so shifts in the ED50 value can be determined (Experiment 2 in Table 3). If the treatment blocks the mechanisms underlying opioid tolerance, then administration of the opioid a day later should produce comparable antinociception with that produced by opioid administration in the opioid-naïve control group.

**Figure 5.**
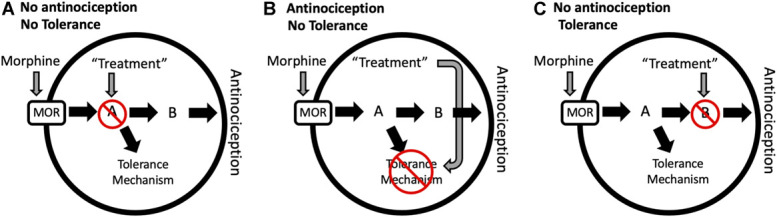
Neuronal signaling model showing the mechanism by which an effective treatment blocks opioid tolerance without blocking antinociception. The binding of morphine or other opioids to the mu-opioid receptor (MOR) triggers a signaling cascade that produces antinociception (path MOR to A to B to antinociception) and tolerance (path MOR to A to Tolerance Mechanism). (A) A putative treatment acting early in the signaling cascade would prevent antinociception and engagement of the tolerance mechanism. (B) A treatment that targets the tolerance mechanism specifically would prevent tolerance without altering the antinociceptive effect of the opioid. (C) A putative treatment that blocks signaling beyond the tolerance mechanism would prevent antinociception without altering the development of tolerance.

Interpretation of the results is difficult even with a good experimental design. The treatment could block the mechanism underlying opioid tolerance or any of the signaling steps preceding this mechanism. As mentioned previously, co-administration of the opioid receptor antagonist naltrexone with morphine will block the development of tolerance by preventing morphine from engaging the mechanism (Fig. 3). The solution to this problem is to systematically test each step of the signaling cascade. A treatment that blocks signaling downstream from the tolerance mechanism will prevent antinociception but will not block tolerance because each opioid injection engages the tolerance mechanism (Fig. 5).

The experimental design for the expression of tolerance is much simpler. Half the animals receive repeated opioid injections to induce tolerance, and half receive repeated vehicle injections as a control. On the last day, all animals are given log-step doses of the opioid. Before opioid administration, half the opioid-treated and half the vehicle-treated animals are given the treatment and the other half are given vehicle (Table 4). This approach will reveal whether the treatment reverses tolerance or produces antinociception or motor effects that could be misinterpreted as blocking tolerance.

**Table 4:** Experimental design to assess the mechanism underlying the expression of opioid tolerance.

Days 1–5 injections, no testing	Day 6 injections and tests
Vehicle	Vehicle/opioid dose-response: assess antinociception
Vehicle	Treatment/opioid dose-response: assess antinociception
Opioid	Vehicle/opioid dose-response: assess antinociception
Opioid	Treatment/opioid dose-response: assess antinociception

Interpretation of experiments examining the expression of tolerance is also much simpler than interpreting the results of experiments examining the development of tolerance. A treatment that blocks the expression of tolerance will enhance antinociception in animals that have received repeated opioid injections. In contrast, putative treatments that block the signaling cascade before or following the tolerance mechanism will block the antinociceptive effect (Fig. 5). This block will be evident in both the animals that received repeated opioid injections and those that are receiving the opioid for the first time.

## 5. Conclusion

Despite extensive research focused on the neural mechanisms of opioid tolerance, a clear understanding remains elusive. Part of the problem is there are multiple mechanisms of opioid tolerance. Moreover, the binding of an opioid to a receptor initiates a signaling cascade that involves multiple molecules and cells. Few studies acknowledge that putative treatments can disrupt signaling anywhere along this pathway without disrupting the mechanism for tolerance specifically. A systematic analysis of each step of this signaling pathway is needed to distinguish the molecular changes that underlie tolerance from treatments that alter opioid antinociception or the assessment of opioid antinociception. Systematic analysis of these signaling pathways is a monumental task, but one that simply relies on good experimental design. This includes using control groups, dose-response analyses, and managing confounds such as repeated testing and antinociceptive and/or motor effects of putative treatments. Our hope is that this review highlighting potential problems and providing solutions will guide future research. Our goal is to identify the key neural mechanisms underlying opioid tolerance from the 101 listed in Table 1, so novel treatments to improve the treatment of pain can be developed.

## Disclosures

The authors have no conflict of interest to declare.
